# Compensatory Cognitive Training for Latino Youth at Clinical High Risk for Psychosis: Study Protocol for a Randomized Controlled Trial

**DOI:** 10.3389/fpsyt.2019.00951

**Published:** 2020-01-10

**Authors:** Zanjbeel Mahmood, Skylar Kelsven, Kristin Cadenhead, Janae Wyckoff, Francisco Reyes-Madrigal, Camilo de la Fuente-Sandoval, Elizabeth W. Twamley

**Affiliations:** ^1^San Diego State University/University of California, San Diego Joint Doctoral Program in Clinical Psychology, San Diego, CA, United States; ^2^Research Service, VA San Diego Healthcare System, San Diego, CA, United States; ^3^Department of Psychiatry, University of California, San Diego, San Diego, CA, United States; ^4^Laboratory of Experimental Psychiatry, Instituto Nacional de Neurología y Neurocirugía, Mexico City, Mexico; ^5^Neuropsychiatry Department, Instituto Nacional de Neurología y Neurocirugía, Mexico City, Mexico; ^6^Center of Excellence for Stress and Mental Health, VA San Diego Healthcare System, San Diego, CA, United States

**Keywords:** cognition, attention, memory, executive functioning, rehabilitation, schizophrenia

## Abstract

**Background:** Early psychosocial interventions targeting cognitive and functional outcomes in individuals at clinical high risk for psychosis are a research priority. An even greater need is the identification of effective interventions in underserved populations. Compensatory Cognitive Training (CCT) is a psychosocial intervention with demonstrated efficacy in chronic schizophrenia and first episode psychosis, but remains to be evaluated in pre-illness phases. The aim of this study was to describe the development and implementation of an ongoing pilot randomized controlled trial investigating the efficacy of group-based, manualized CCT, as compared to recreational therapy (RT), for Latino participants at clinical high risk for psychosis (CHR) in both the United States and Mexico. It is hypothesized that, in comparison to those receiving RT, participants receiving CCT will show significant improvements in neurocognitive performance and functional capacity (co-primary outcomes) and self-rated functioning and clinical symptoms (secondary outcomes).

**Methods:** Latino CHR participants aged 12–30 years will be included in the study. Both CCT and RT will be delivered in either Spanish or English, depending on group preference. Additionally, all assessments will be administered in participants’ preferred language. A comprehensive assessment of neurocognitive and functional performance and clinical symptomatology will be performed at baseline, mid-intervention (4 weeks, 8 weeks), post-intervention (12 weeks) and 3-month follow-up. The primary outcome measures are neurocognition and functional capacity, as assessed by the MATRICS (Measurement and Treatment Research in Cognition in Schizophrenia) Consensus Cognitive Battery and the University of California, San Diego Performance-Based Skills Assessment-Brief, respectively. Furthermore, secondary outcomes measures will be used to examine change in clinical symptoms and self-reported functioning in response to CCT versus RT.

**Discussion:** The evaluation of a novel treatment such as CCT in CHR youth will provide empirical support for a low risk, comprehensive cognitive intervention that could have important implications for public health if it improves neurocognition and functioning.

## Introduction

Recent efforts to extend medical prevention models to the field of schizophrenia have resulted in systematic, reliable identification of individuals who are at clinical high risk (CHR) for imminent onset of psychosis (i.e., putative prodromal psychosis) (1, 2). Compared to psychotic disorders, the symptoms of psychosis-risk syndrome are less severe and more transient. Symptoms are not longstanding trait-pathology, but rather present as a marked change in individual’s mental state, evidenced through report from self and concerned others. Specific subtypes of the psychosis-risk syndrome are identified through formal structured clinical interview and are differentiated through individual risk factors. Broadly, risk factors for psychosis-risk syndrome include presence of: attenuated positive symptoms, negative symptoms, cognitive impairment or cognitive decline, decline in social, and/or role functioning, as well as family history of psychosis (3, 4). Diagnosis of a psychosis-risk syndrome requires that symptoms are associated with functional impairment and/or distress and of recent onset or worsening.

Findings from the North American Prodrome Longitudinal Study (NAPLS) have established that within the first 1–2 years after formal identification of being at CHR for psychosis, 20–40% of individuals go on to develop an acute psychotic illness (3, 5, 6). Despite advances in the early identification of individuals meeting CHR criteria, there is significant need for effective interventions that can be implemented early in the course of illness to improve symptoms and functional outcomes, especially considering that the strongest predictors of conversion to psychotic illness include modifiable risk factors such as prodromal symptom severity (as measured through structured interview), declines in social functioning, as well as verbal learning and memory deficits (6). Namely, there is a need for interventions that target the emerging neurocognitive (7–9), information processing (10–12), social, role, and global functioning deficits (13, 14) that characterize the prodromal period of illness. Not only are these early deficits disruptive to normal development and life trajectories, but they are also predictive of later conversion to psychotic illness (6, 15).

Antipsychotic medication, although effective in controlling positive symptoms, does not ameliorate cognitive deficits (16), nor has it shown any effects on conversion in randomized clinical trials (17, 18). Moreover, ethical concerns regarding exposing young people to psychotropic agents when less than half (~35%) are expected to convert to psychosis (19, 20) further support “staging” the prodromal period much like other medical illnesses (e.g., cancer, diabetes) and using less invasive treatments (psychosocial, education) for the early stages. Neurocognitive deficits are present across all identified stages of the CHR state and remain relatively stable, even during symptomatic remission upon illness onset (21). As such, an individualized, low risk treatment algorithm that focuses on neurocognition and functioning, in addition to the presenting subsyndromal psychotic symptoms, is a logical intervention strategy for this phase of the illness.

To date, there have been a limited number of randomized controlled trials analyzing the effectiveness of various psychosocial interventions in CHR, such as cognitive behavioral therapy (CBT), family focused therapy (FFT), or cognitive remediation (5, 22–24). Meta-analyses of psychosocial treatment effects on attenuated psychotic symptoms and negative symptoms yielded no statistically significant treatment effects for any intervention examined, although there were trends (p = .07) for CBT, FFT, and cognitive remediation (25, 26). Importantly, the existing RCTs use “transition to psychosis” as the primary outcome measure of treatment efficacy. However, the goal of psychosocial treatments may be reframed as improving cognitive, social, and functional impairments that are associated with poor prognosis in CHR, rather than preventing conversion to psychosis. In other words, for some individuals, “transition to psychosis” may be inevitable, but the degree of impairment associated with psychosis can be reduced if these individuals are provided skills to better cope with the cognitive and functional deficits associated with CHR.

In fact, cognitive deficits are a key determinant of functional outcomes in people with schizophrenia (27, 28) as well as individuals meeting CHR criteria (29, 30), irrespective of later development of a psychotic illness (15, 31, 32). Thus, there is a critical need for early interventions to improve cognitive impairment (and, therefore, everyday functioning) rather than focusing solely on symptomatic remission. Cognitive intervention trials (i.e., cognitive remediation or cognitive training) in individuals meeting CHR or early psychosis criteria remain limited and have produced mixed findings (33); although improvements in cognitive outcomes have been noted in some cognitive domains, these cognitive gains do not always generalize to improvements in community functioning. Although pro-cognitive interventions in pre- and early illness stages appear promising, preliminary evidence suggests that a compensatory strategy approach may best target areas of cognition typically impaired in early psychosis (34).

Compensatory Cognitive Training (CCT; (35–37), a strategy-based cognitive training approach, is one such psychosocial intervention that may hold promise as an efficacious treatment for CHR individuals. The most recent review and meta-analysis of cognitive remediation studies (35) found the largest effect sizes for compensatory strategy-based approaches in the context of psychiatric rehabilitation. In addition to improvements in neurocognitive performance and functional outcomes, CCT has demonstrated a large effect size for negative symptoms (38, 39). CCT strategies teach participants how to bypass their deficits and directly address functional recovery through a focus on application of appropriate cognitive strategies in the real world. In essence, CCT provides an intervention that targets healthy neural circuitry to compensate for damaged circuit elements, or even protect this circuitry from future damage (40). The merits of CCT also exist in its format; it is manualized, group-based, low-tech, brief (38), and can easily be applied in the community in English or Spanish, making it a practical intervention for underserved populations.

Latino CHR individuals represent an underserved population in the United States. Latinos have become the largest minority group in the US (41); more than half (~54%) of California’s elementary children are now of Latino origin (42). Despite the rising population of Latinos, disparities in mental health care continue to exist (43). Availability of and access to mental health resources is also limited in Mexico, with the Instituto Nacional de Neurología y Neurocirugía (INNN) serving as a local and national reference institution for the evaluation of CHR and early psychosis cases in Mexico City, a catchment area of over 20 million people. Latinos with CHR also present unique clinical challenges, presenting greater educational needs and exhibiting more severe negative symptoms predictive of conversion to psychosis (44).

Considering negative symptom severity is a predictor of long-term poor psychosocial functioning in schizophrenia (45) and CHR patients (30), and the lack of efficacy of any psychosocial or pharmacologic interventions in improving this symptom dimension in CHR patients (26), CCT’s demonstrated efficacy for improving both cognition and negative symptom severity makes it a suitable intervention to evaluate within Latino CHR youth. As such, the aim of this paper is to describe the development and implementation of an ongoing pilot randomized controlled trial investigating the efficacy of CCT, as compared to recreational therapy (RT), for CHR Latino participants in both the United States and Mexico. We hypothesize that, in comparison to those receiving RT, participants receiving CCT will show significant improvements in neurocognitive performance and functional capacity (co-primary outcomes) and self-rated functioning and clinical symptoms (secondary outcomes).

## Design and Methods

We are currently conducting a dual center (University of California, San Diego [UCSD] and INNN) randomized controlled trial of CCT compared to RT for CHR Latino youth in the United States and Mexico. The study is registered as a clinical trial (Clinical Trials registration number: NCT02245607). Baseline assessment confirms CHR criteria, and participants are subsequently randomized in groups to receive group-based CCT or RT. Study procedures were approved by the University of California, San Diego Institutional Review Board and by the Ethics and Scientific Committees of INNN.

### Participants

Projected enrollment is 120 CHR Latino youth (60 participants per site). In San Diego, participants are referred to the study by community health practitioners in San Diego who serve the Latino community, public schools in high Latino districts, County Mental Health, and the National Alliance on Mental Illness. In Mexico City, participants are recruited *via* the Neuropsychiatric Service, Early Psychosis Clinic, an advocacy group (Asociación de Familiares y Amigos de Personas con Esquizofrenia - AFAPE), and the Adolescent Study of Neuropsychiatric Assessment and Imaging (PIENSA) Program at INNN. Inclusion criteria are: 1) between the ages of 12 and 30; 2) meet CHR criteria per the Structured Interview for Prodromal Syndromes (SIPS); and 3) be of Latino descent. Specific to the Mexico City site, participants are eligible only if Spanish is their preferred language. Exclusionary criteria are: 1) current or lifetime psychotic disorder; 2) concomitant medical or neurological illness; 3) brain injury with loss of consciousness >30 min; 4) current substance abuse that interferes with group or assessment procedures or is judged to be causing subsyndromal psychotic symptoms (excluding nicotine); 5) IQ < 80; 6) high suicidal risk; and 7) Axis I disorder or substance use that better accounts for subsyndromal psychotic symptoms. Latino descent was identified through participant/family self-report. Only Spanish-speaking individuals were eligible for enrollment at the INNN site to reduce confounding factors associated with using two separate manuals presented in different languages for groups at that site. Psychotropic medication is permitted, including antipsychotics, antidepressants, and mood stabilizers. All cases are discussed in a weekly conference call between sites to reach consensus on inclusion/exclusion criteria and symptom and functional ratings. All participants over the age of 18 provide written informed consent; minors provide assent with written consent by a parent or legal guardian.

### Treatment

Eligible participants are assigned to one of the two treatment conditions: CCT and RT. Both treatments are manualized, 12-week interventions. Via a randomization schedule developed by the study statistician, all eligible participants are randomized in groups (goal 4–6 per group) to receive 12 weeks of CCT or RT. Moreover, participants’ age is factored in the randomization process to ensure that each intervention group only includes participants falling within a distinct age group (12–14, 15–17, and 18–30). This guideline was determined through direct communication with the National Institute of Mental Health (NIMH), given NIMH’s concern for minors being co-enrolled and randomized into the same group with legal-aged adults.

#### Compensatory Cognitive Training

CCT is delivered in accordance with the manual and has been described extensively elsewhere (38). Briefly, participants in the CCT group receive weekly 90-min group CCT sessions for 12 weeks, which involve education regarding targeted skill areas as well as modules of compensatory strategies targeting prospective memory, attention, learning/memory, and executive functioning. The CCT strategies are presented in an interactive, game-like format to maintain interest and reduce attrition. Homework assignments are designed to encourage additional practice outside treatment sessions. At the beginning of each session, previous homework assignments are reviewed or completed *in vivo* to ensure skill proficiency and execution. As the ongoing study is a feasibility trial, minor age-related modifications were made as necessary to ensure CCT was engaging and relevant to the speciﬁc needs of young Latinos with CHR (e.g., using Spanish names, focusing on school-related versus work-related examples).

English and Spanish versions are used in the study and employed based on the group’s preferred language. The Spanish translation of the CCT manual (46), a collaborative effort between investigators at UCSD, INNN, and the University of Deusto, Bilbao, is applicable in both Spain and Latin America (translation performed by natives of Spain and Mexico). Translation back to English by translators unfamiliar with the manual was very successful and the final Spanish CCT manual required very little modification.

#### Recreational Therapy

RT was selected as a robust control condition, a group therapy intervention that provides the same frequency and amount of therapist and other group member contact as CCT, but does not provide any cognitive training. Participants assigned to the RT sessions participate in different recreational activities targeting their popular culture awareness, art, and physical activities. The 12 sessions of RT, also broken down into modules, consist of discussions on topics such as music, current events, art, and health. Reference materials were selected from current media (newspapers, magazines, and internet). Practice of RT skills is encouraged outside of session, but no formal homework assignments are given. At the beginning of each session, previous week’s concepts are reviewed. The purpose of these sessions is to provide participants with access to information that would encourage social interactions and the addition of physical activity to their daily routine.

### Assessments

Following enrollment/randomization, participants complete assessments at baseline, mid-intervention (4 weeks, 8 weeks), post-intervention (12 weeks) and 3-month follow-up, conducted by clinical raters and research assistants blind to group assignment (see **Table 1** for the Assessment Overview). Participants are compensated for their time per assessment and at each group session to offset the cost of transportation. Following baseline assessment, participants receive 12 weeks of their assigned intervention, delivered by bachelor’s level or above therapists at each site. Therapists administer both treatments in an alternating schedule to avoid therapist effects in the design. The primary outcome measures are neurocognition and functional capacity, as assessed by the measures detailed below. Furthermore, secondary outcomes measures will be used to examine change in clinical symptoms and self-reported functioning in response to CCT versus RT.

**Table 1:** Assessment Overview.

	Time (Hour : Min)	Baseline	Week 4	Week 8	Week 12	Week 24
**Clinical (Day 1)**	**2:30**	**X**			**X**	**X**
Demographics	:15	**X**			**X**	**X**
SCID-I	1:00	**X**			**X**	**X**
SIPS/SOPS	1:00	**X**	**X**	**X**	**X**	**X**
AUS/DUS	:15	**X**	**X**	**X**	**X**	**X**
**Functional (Day 1)**	**1:35**	**X**			**X**	**X**
SAS-SR	:15	**X**			**X**	**X**
GAF	:05	**X**			**X**	**X**
SLoF	:30	**X**			**X**	**X**
UPSA	:45	**X**			**X**	**X**
***Day 1 Total***	***4:05***	**X**			**X**	**X**

**Neurocognitive** **Battery (Day 2)**	**2:15**	**X**			**X**	**X**
**General Intelligence**						
WAIS Vocabulary	:15	**X**			**X**	**X**
WAIS Block Design	:10	**X**			**X**	**X**
**Verbal Learning**						
HVLT	:05	**X**			**X**	**X**
BVMT	:05	**X**			**X**	**X**
**Processing Speed**						
BACS Symbol Coding	:05	**X**			**X**	**X**
Category Fluency	:05	**X**			**X**	**X**
Trails A	:05	**X**			**X**	**X**
**Attention/Vigilance**						
CPT-IP	:10	**X**			**X**	**X**
**Working Memory**						
WMS III Spatial Span	:10	**X**			**X**	**X**
LNS	:10	**X**			**X**	**X**
**Executive Functioning**						
NAB Mazes	:10	**X**			**X**	**X**
WCST	:10	**X**			**X**	**X**
***Day 2 Total***	***2:15***	**X**			**X**	**X**

***Exit Interview***	***:30***				**X**	
**Total Assessment Time** (H:M)		**6:20**	**1:15**	**1:15**	**6:50**	**6:20**

Structured Interview for Prodromal Syndromes (SIPS), Scale of Prodromal Symptoms (SOPS), Structured Clinical Interview for DSM-IV (SCID) Axis I, The Social Adjustment Scale – Self-Report (SAS-SR), Global Assessment of Functioning (GAF), Specific Level of Functioning (SLoF), The UCSD Performance-Based Skills Assessment (UPSA), Wechsler Adult Intelligence Scale (WAIS), Hopkins Verbal Learning Test (HVLT), Brief Visual Memory Test–R (BVMT), Brief Assessment of Cognition in Schizophrenia (BACS), Category Fluency, Trail Making Part A, Continuous Performance Task Identical Pairs (CPT-IP), Wechsler Memory Scale (WMS), Univ. of Maryland Letter-Number Span (LNS), Neuropsychological Assessment Battery (NAB) Mazes, Wisconsin Card Sorting Test (WCST).

#### Cognitive and Functional Assessment

Premorbid intellectual functioning is assessed *via* the Wechsler Adult Intelligence Scale (WAIS), Block Design and Vocabulary subtests. Participants are administered an expanded MATRICS (Measurement and Treatment Research in Cognition in Schizophrenia) Consensus Cognitive Battery (MCCB) in English or Spanish (47). The MCCB has excellent qualities, including psychometric properties for longitudinal studies, utility as a repeated measure, relevance to functional outcome, brevity, ease of use, and participant tolerability. These tests are administered according to published standardized procedures. As part of collaborative studies, the UCSD team has traveled to Mexico City and standardized administration of the neuropsychological battery. The battery includes the following domains: 1) Estimated IQ: Wechsler Adult Intelligence Scale Vocabulary and Block Design, 2) Learning & Memory: Hopkins Verbal Learning Test, Brief Visual Memory Test–R, 3) Processing Speed: Brief Assessment of Cognition in Schizophrenia: Symbol Coding, Category Fluency, Trail Making Part A, 4) Attention/Vigilance: Continuous Performance Task Identical Pairs, 5) Working Memory: WMS III Spatial Span, University of Maryland Letter-Number Span, and 6) Executive Functioning: Neuropsychological Assessment Battery Mazes, Wisconsin Card Sorting Test. A Global Cognitive Index will combine z scores of all cognitive domains per established methods (8). The University of California, San Diego Performance-Based Skills Assessment-Brief (UPSA-Brief) was selected to measure performance-based functional capacity (48), along with UPSA-Child and Adolescent version for adolescents. The Specific Levels of Functioning (SLOF) scale is used as a self-reported functioning measure because of its concordance with objective ability measures (49). In addition, the modified Global Assessment of Functioning (50) and the Social Adjustment Scale (51) are administered.

#### Clinical Assessment

CHR is assessed using the SIPS, with symptom ratings measured via the Scale of Prodromal Symptoms (SOPS) (1). To assure reliable diagnostic and clinical assessment across sites, a consensus diagnosis procedure was developed, including weekly clinical calls with certified Ph.D. or M.D. raters, all of whom were trained by Yale developers of the SIPS. Participants are also administered Structured Clinical Interview for DSM-IV Axis I Disorders (SCID) (52). Current and past psychosocial treatment, medication and hospitalization data are collected for all participants. Finally, the Alcohol/Drug Use Scale (AUS/DUS) is administered to measure current substance use (53).

During the final group session, all participants are given an exit interview in which they complete a small survey examining overall satisfaction with the intervention. Participants are asked to provide self-rated impressions of improvement in concentration, memory, attention, as well as conversational and task vigilance. These impressions are rated on a scale of 1 (not at all helpful) to 10 (very helpful) referring to whether participants found the skills taught during group to be helpful in improving each respective domain of cognition. Participants are also given the opportunity to provide qualitative feedback regarding additional topics to be covered, topics to be removed, and feedback regarding difficulty attending group sessions, as well as any other suggestions to improve groups.

### Design Considerations

Focusing on compensatory and environmental strategies, rather than drill and practice computer exercises, is consistent with empirical focus on improving functional skills among patients with schizophrenia (54, 55). In addition to attention, learning and memory, and executive functioning, the CCT intervention targets prospective memory ability (i.e., the ability to remember to do things in the future, such as complete homework assignments or attend a doctor’s appointment). These CCT-targeted areas of cognition represent potentially modifiable cognitive domains with relevance for psychosocial functioning (27, 56, 57). Moreover, these domains represent areas of cognition also affected in the prodromal phase (9, 58) and predictive of later conversion to psychosis, thereby serving as key initial treatment targets. Prevention of further deterioration and preservation of cognitive abilities may be vital first steps to increase the effectiveness of other psychosocial treatments. Another strength of CCT is its exploitation of stronger cognitive functions in schizophrenia, such as imagery (59) and habit learning (60, 61), to bolster impaired abilities; for example, forming new habits in attention and problem-solving can lead to gains in performance efficiency *via* automatic processing and decreased cognitive demands. Increasing individuals’ ability to remember appointments, sustain attention, encode important concepts, and think flexibly may well improve the success of concomitant treatments.

### Therapist Training

Mental health providers at the bachelor’s level or above deliver the treatment. Two San Diego site therapists, as well as one investigator and one study therapist at the Mexico City site, were formally trained on the CCT protocol by EWT at study initiation. The 2-day training included an introduction to the theoretical principles underpinning the treatment model, specific instructions on implementing each of the twelve CCT sessions, and review of RT sessions. Weekly individual supervision is ongoing throughout the trial between the study therapists and PIs.

### Fidelity

Research recommendations by Perepletchikova and Kazdin (62) were implemented to maximize treatment manual adherence (e.g., use of checklists). All CCT and RT sessions are recorded to monitor fidelity; sessions are rated using items from the Cognitive Training Fidelity Scale (unpublished; available upon request from the authors). Therapist compliance was defined as 90% adherence to the items on the weekly checklist; subthreshold fidelity ratings will result in remedial training until these levels are achieved. To reduce the risk of treatment contamination, groups will be held at times and locations where subjects in different groups will not have the opportunity to meet in a waiting room. We will also ask subjects not to discuss their treatment with other subjects until after completion of the protocol. Monthly fidelity ratings will be fed back to therapists during supervision to improve fidelity. RT sessions will also be rated to ensure that RT groups do not receive training in CCT skills.

### Data Analysis

The purpose of this pilot RCT is to examine feasibility and generate effect sizes to establish benchmarks for future studies, not to complete an adequately powered efficacy study. Power calculations based on Cohen method (63) and the method provided by Hedeker et al. for the Random Regression Model (64) indicated that with the proposed sample size, we will have a minimum 80% power to detect a medium to large effect across groups, consistent with the medium to large effects in prior studies.

A linear mixed model, with post-hoc procedures if indicated, will be used to analyze data in order to compare CCT versus RT in Latino CHR subjects in the United States and Mexico (co-primary outcomes: neurocognition [Global Cognitive Index and individual domain scores] and functional capacity; secondary outcomes: self-reported functioning and clinical symptom severity). Feasibility data such as recruitment rate, consent rate, reasons for not participating, reasons for dropping out, participants’ satisfaction, and their suggestions to improve the study will be tabulated and will be analyzed descriptively.

Furthermore, predictors (moderators) of response to CCT versus RT, including age, baseline symptom severity, neurocognition, and comorbidity, will be explored. Moderators will be examined by building hierarchical linear models with potential moderator variables (e.g., baseline symptoms, neurocognition, functioning, age, and comorbidity) included in the model (65). The linear model to be used for both moderator and mediator analyses will compare CCT versus RT treatment groups. The independent variables are treatment group, moderator, and the treatment-moderator interaction. An interactive effect will mean that the effect of treatment on individual subjects depends on their value of moderator.

Finally, mediators of functional outcomes, including improvement in cognition and symptom severity, will be explored. Mediation analyses will use a similar linear mixed models approach. Two conditions must be met for mediation of the treatment effect: 1) correlation between the mediator and treatment; and 2) relationship between the mediator and outcome (65). First, we will test the effect of treatment group and the group × time interaction on the mediator (for example, improvement in cognition), and we expect a statistically significant group × time interaction. Second, we will test the effects of neurocognitive change (change score from baseline to mid-treatment) and the Global Cognitive Index change × group interaction on the outcome (UPSA) in the model that includes group and time, and we expect a statistically significant mediator × time or mediator × group × time interaction. The Global Cognitive Index change score from baseline to midway establishes temporal precedence of the mediator. We will also explore whether change in Global Cognitive Index and other variables mediates change in the other outcome variables (e.g., functioning). Finally, we will explore the relationships between specific measures (e.g., specific symptom factors) and functioning, number of sessions attended, as well as relationships between change in one domain and change in another (e.g., between change in symptoms, neurocognition, with change in functioning), when appropriate.

## Discussion

Developing psychosocial interventions to improve cognitive and functional outcomes in CHR participants is a research priority. In addition, the development of treatments that can be feasibly and acceptably delivered to diverse and underserved populations is greatly needed. CCT is a psychosocial intervention with demonstrated efficacy in first episode and chronic schizophrenia (35, 36), but remains to be evaluated in pre-illness phases.

Studies of cognitive training interventions in pre-psychotic illness remain limited. One study has demonstrated acceptability and feasibility of a compensatory-based approach (Cognitive Adaptive Training) in early illness phase (34); however, the small sample size (*n* = 5), individual format, and lengthy duration undermined its widespread application in real-world settings. Although preliminary evidence suggests computerized drill-and-practice as a feasible intervention with potential cognitive benefits for CHR (66), controlled studies are not yet published. The availability of CCT as a freely accessible, brief, manualized, group-based intervention provides a low-cost, scalable, and clinically relevant treatment with promise for widespread uptake and implementation. CCT’s utility is further boosted by its availability in both English and Spanish and because it can be delivered by bachelor and master’s level clinicians. Furthermore, sample diversity in the ongoing trial is enhanced *via* recruitment from both US and Mexico, demonstrating attention to burgeoning national and international preventive efforts in psychosis research.

Several aspects of our study design could affect feasibility of participant recruitment and retention across sites, as well as generalizability. First, due to the nature of our established clinical services, groups are facilitated outpatient psychiatric service settings rather than primary care. In the United States, minority/immigrant individuals may be more likely to engage in mental health treatment through primary care settings as compared to specialty mental health settings (67, 68). Second, to increase socialization, we opted to offer clinic-based groups rather than home-based care. Some participants who prefer individual treatment or who cannot easily access transportation may be less likely to participate. The willingness of parents of minors to take time off work to facilitate their children’s participation may also affect participation and retention. As such, future investigations may consider CCT delivery in real-world settings, or a telemedicine approach. To our knowledge, no studies to date have sought to investigate the feasibility of remote treatment delivery methods for CHR populations. Third, group randomization and stratification methods require that children, adolescents, and adults be separated into different groups. Thus, additional challenges are introduced in recruiting a sufficient number of individuals within the same age cohort to begin a group of at least 4–6 individuals. Finally, our results may not generalize to non-Latino CHR youth. Research on Latino youth indicate that prevalence rates of depressive symptomatology and alcohol use are significantly higher in Latino youth and these populations have inadequate access to mental health services (69). Thus, in addition to the considerations above, it is possible that CCT is differentially effective for this population given known mental health co-morbidities as well as access barriers.

Despite these potential limitations in study design, the introduction of cognitive training techniques at the INNN, the primary psychosis referral center in a city of over 20 million, is significant in that very few psychosocial treatments for schizophrenia are administered in Mexico. This work will clearly influence the treatment of psychotic illness in Latin America and the United States. This study will provide valuable information regarding the feasibility and efficacy of CCT to treat CHR Latino youth. UCSD’s association with the North American Prodrome Longitudinal Studies (NAPLS) Consortium and the International Prodromal Research Network offers a platform for a larger scale treatment study should the proposed treatment prove promising. If found to be efficacious, CCT-associated improvements in cognitive and functional performance may well enhance success of concomitant treatments.

## Ethics Statement

The studies involving human participants were reviewed and approved by University of California, San Diego Institutional Review Board Ethics and Scientific Committees of Instituto Nacional de Neurología y Neurocirugía (INNN). Written informed consent to participate in this study was provided by the participants’ legal guardian/next of kin.

## Author Contributions

The Principal Investigators (CF-S, ET, and KC) were involved in designing and implementing the study. ZM and SK were primarily responsible for manuscript preparation. ET, KC, FR-M, and CF-S were involved in development, preparation, and distribution of the English and Spanish versions of the Compensatory Cognitive Training (CCT) manual. JW and FR-M served as group facilitators for the trial. Further, ET and FR-M served as clinical supervisors for CCT group facilitators. FR-M was involved in group facilitation and assessment procedures. All authors were involved in editing the manuscript and approved its final content.

## Funding

Data for this study was collected as part of a National Institutes of Mental Health Grant R34 MH105247 (co-PIs ET, KC, and CF-S). CF-S is supported by CONACyT’s Sistema Nacional de Investigadores.

## Conflict of Interest

CF-S has served as a consultant for Janssen, and FR-M has served as a speaker for AstraZeneca.

The remaining authors declare that the research was conducted in the absence of any commercial or financial relationships that could be construed as a potential conflict of interest.

## References

[1] Miller TJ McGlashan TH Rosen JL Cadenhead K Ventura J McFarlane W Prodromal assessment with the structured interview for prodromal syndromes and the scale of prodromal symptoms: predictive validity, interrater reliability, and training to reliability. Schizophr Bull (2003) 29:703–16. 10.1093/oxfordjournals.schbul.a007040 14989408

[2] Yung A Phillips L McGorry P Ward J Donovan K Thompson K Comprehensive assessment of at-risk mental states (CAARMS). Melbourne, Australia, University of Melbourne, Department of Psychiatry, Personal Assessment and Crisis Evaluation Clinic. (2002).

[3] Fusar-poli P Bonoldi I Yung AR Borgwardt S Kempton MJ Valmaggia L Predicting psychosis: meta-analysis of transition outcomes in individuals at high clinical risk. Arch Gen Psychiatry (2012) 69:220–9. 10.1001/archgenpsychiatry.2011.1472 22393215

[4] McGlashan T Walsh BC Woods SW The Psychosis Risk Syndrome: Handbook for Diagnosis and Follow-up. Oxford University Press: New York: New York (2010).

[5] Fusar-Poli P Borgwardt S Bechdolf A Addington J Riecher-Rössler A Schultze-Lutter F The psychosis high-risk state: a comprehensive state-of-the-art review. Arch Gen Psychiatry (2013) 70:107–20. 10.1001/jamapsychiatry.2013.269 PMC4356506 23165428

[6] Cannon TD Cadenhead K Cornblatt B Woods SW Addington J Walker E Prediction of psychosis in youth at high clinical risk: a multisite longitudinal study in North America. Arch Gen Psychiatry (2008) 65:28–37. 10.1001/archgenpsychiatry.2007.3 18180426 PMC3065347

[7] Jahshan CMS Heaton RK Golshan S Cadenhead KS Course of neurocognitive deficits in the prodrome and first episode of schizophrenia. Neuropsychology (2010) 24:109–20. 10.1037/a0016791 PMC2808194 20063952

[8] Eastvold AD Heaton RK Cadenhead KS Neurocognitive deficits in the (putative) prodrome and first episode of psychosis. Schizophr Res (2007) 93:266–77. 10.1016/j.schres.2007.03.013 PMC2080673 17467955

[9] Seidman LJ Giuliano AJ Meyer EC Addington J Cadenhead KS Cannon TD Neuropsychology of the prodrome to psychosis in the NAPLS consortium. Arch Gen Psychiatry (2010) 67:578–88. 10.1001/archgenpsychiatry.2010.66 PMC3332118 20530007

[10] Cadenhead KS Light GA Shafer KM Braff DL P50 suppression in individuals at risk for schizophrenia: the convergence of clinical, familial, and vulnerability marker risk assessment. Biol Psychiatry (2005) 57:1504–9. 10.1016/j.biopsych.2005.03.003 15953486

[11] Quednow BB Frommann I Berning J Kuhn KU Maier W Wagner M Impaired sensorimotor gating of the acoustic startle response in the prodrome of schizophrenia. Eur Psychiatry (2008) 23:S57.04. 10.1016/j.biopsych.2008.04.019 18514166

[12] Mondragón-Maya A Solís-Vivanco R León-Ortiz P Rodríguez-Agudelo Y Yáñez-Téllez G Bernal-Hernández J Reduced P3a amplitudes in antipsychotic naïve first-episode psychosis patients and individuals at clinical high-risk for psychosis. J Psychiatr Res (2013) 47:755–61. 10.1016/j.jpsychires.2012.12.017 23507048

[13] Ballon JS Kaur T Marks II Cadenhead KS Social functioning in young people at risk for schizophrenia. Psychiatry Res (2007) 151:29–35. 10.1016/j.psychres.2006.10.012 17383739 PMC3065359

[14] Cornblatt BA Auther AM Niendam T Smith CW Zinberg J Bearden CE Preliminary findings for two new measures of social and role functioning in the prodromal phase of schizophrenia. Schizophr Bull (2007) 33:688–702. 10.1093/schbul/sbm029 17440198 PMC2526147

[15] Cornblatt BA Carrión RE Addington J Seidman L Walker EF Cannon TD Risk factors for psychosis: impaired social and role functioning. Schizophr Bull (2012) 38:1247–57. 10.1093/schbul/sbr136 PMC3494064 22080497

[16] Rund BR Borg NE Cognitive deficits and cognitive training in schizophrenic patients: a review. Acta Psychiatr Scand (1999) 100:85–95. 10.1111/j.1600-0447.1999.tb10829.x 10480194

[17] McGlashan TH Zipursky RB Perkins D Addington J Miller T Woods SW Randomized, double-blind trial of olanzapine versus placebo in patients prodromally symptomatic for psychosis. Am J Psychiatry (2006) 163:790–9. 10.1176/ajp.2006.163.5.790 16648318

[18] McGorry PD Nelson B Phillips LJ Yuen HP Francey SM Thampi A Randomized controlled trial of interventions for young people at ultra-high risk of psychosis: twelve-month outcome. J Clin Psychiatry (2013) 74:349–56. 10.4088/JCP.12m07785 23218022

[19] Haroun N Dunn L Haroun A Cadenhead KS Risk and protection in prodromal schizophrenia: Ethical implications for clinical practice and future research. Schizophr Bull (2006) 32:166–78. 10.1093/schbul/sbj007 PMC2632176 16207892

[20] Nelson B Yuen HP Wood SJ Lin A Spiliotacopoulos D Bruxner A Long-term follow-up of a group at ultra high risk (“Prodromal”) for psychosis: the PACE 400 study. JAMA Psychiatry (2013) 70:793–802. 10.1001/jamapsychiatry.2013.1270 23739772

[21] Bozikas VP Andreou C Longitudinal studies of cognition in first episode psychosis: a systematic review of the literature. Aust N Z J Psychiatry (2011) 45:93–108. 10.3109/00048674.2010.541418 21320033

[22] Stafford MR Jackson H Mayo-Wilson E Morrison AP Kendall T Early interventions to prevent psychosis: systematic review and meta-analysis. BMJ (2013) 346:1–13. 10.1136/bmj.f185 PMC3548617 23335473

[23] Thompson E Millman ZB Okuzawa N Mittal V Devylder J Skadberg T Evidence-based early interventions for individuals at clinical high risk for psychosis: a review of treatment components. J Nerv Ment Dis (2015) 203:342–51. 10.1097/NMD.0000000000000287 25919384

[24] Glenthøj LB Hjorthøj C Kristensen TD Davidson CA Nordentoft M The effect of cognitive remediation in individuals at ultra-high risk for psychosis: a systematic review. NPJ Schizophr (2017) 3:1–7. 10.1038/s41537-017-0021-9 28560266 PMC5441569

[25] Devoe DJ Farris MS Townes P Addington J Attenuated psychotic symptom interventions in youth at risk of psychosis: a systematic review and meta-analysis. Early Interv Psychiatry (2019) 13:3–17. 10.1111/eip.12677 29749710 PMC6230498

[26] Devoe DJ Peterson A Addington J Negative symptom interventions in youth at risk of psychosis: a systematic review and network meta-analysis. Schizophr Bull (2018) 44:807–23. 10.1093/schbul/sbx139 PMC6007754 29069511

[27] Green MF Kern RS Braff DL Mint J Neurocognitive deficits and functional outcome in schizophrenia: are we measuring the “right stuff”? Schizophr Bull (2000) 26:119–36. 10.1093/oxfordjournals.schbul.a033430 10755673

[28] Green MF Kern RS Heaton RK Longitudinal studies of cognition and functional outcome in schizophrenia: implications for MATRICS. Schizophr Res (2004) 72:41–51. 10.1016/j.schres.2004.09.009 15531406

[29] Eslami A Jahshan C Cadenhead KS Disorganized symptoms and executive functioning predict impaired social functioning in subjects at risk for psychosis. Arch Gen Psychiatry (2011) 23:457–60. 10.1176/jnp.23.4.jnp457 PMC4113423 22231319

[30] Meyer EC Carrión RE Cornblatt BA Addington J Cadenhead KS Cannon TD The relationship of neurocognition and negative symptoms to social and role functioning over time in individuals at clinical high risk in the first phase of the north american prodrome longitudinal study. Schizophr Bull (2014) 40:1452–61. 10.1093/schbul/sbt235 PMC4193704 24550526

[31] Tarbox SI Addington J Cadenhead KS Cannon TD Cornblatt BA Perkins DO Functional development in clinical high risk youth: prediction of schizophrenia versus other psychotic disorders. Psychiatry Res (2014) 215:52–60. 10.1016/j.psychres.2013.10.006 24200216 PMC3946851

[32] Addington J Cornblatt BA Cadenhead KS Cannon TD McGlashan TH Perkins DO At clinical high risk for psychosis: outcome for nonconverters. Am J Psychiatry (2011) 168:800–5. 10.1176/appi.ajp.2011.10081191 PMC3150607 21498462

[33] Pantelis C Wannan C Bartholomeusz CF Allott K McGorry PD Cognitive intervention in early psychosis - preserving abilities versus remediating deficits. Curr Opin Behav Sci (2015) 4:63–72. 10.1016/j.cobeha.2015.02.008

[34] Allott KA Killackey E Sun P Brewer WJ Velligan DI Feasibility and acceptability of cognitive adaptation training for first-episode psychosis. Early Interv Psychiatry (2016) 10:476–84. 10.1111/eip.12207 25496290

[35] Wykes T Huddy V Cellard C Mcgurk SR Czobor P A meta-analysis of cognitive remediation for schizophrenia: methodology and effect sizes. Am J Psychiatry (2011) 168:472–85. 10.1176/appi.ajp.2010.10060855 21406461

[36] Mendella PD Burton CZ Tasca GA Roy P St. Louis L Twamley EW Compensatory cognitive training for people with first-episode schizophrenia: results from a pilot randomized controlled trial. Schizophr Res (2015) 162:108–11. 10.1016/j.schres.2015.01.016 25631454

[37] Twamley EW Thomas KR Burton CZ Vella L Jeste DV Heaton RK Compensatory cognitive training for people with severe mental illnesses in supported employment: a randomized controlled trial. Schizophr Res (2017) 203:41–8. 10.1016/j.schres.2017.08.005 PMC5816728 28823720

[38] Twamley EW Vella L Burton CZ Heaton RK Jeste DV Compensatory cognitive training for psychosis: effects in a randomized controlled trial. J Clin Psychiatry (2012) 73:1212–9. 10.4088/JCP.12m07686 PMC3593661 22939029

[39] Mahmood Z Clark JMR Twamley EW Compensatory cognitive training for psychosis: effects on negative symptom subdomains. Schizophr Res (2018) 204:397–400. 10.1016/j.schres.2018.09.024 30293693 PMC6402965

[40] Swerdlow NR Are we studying and treating schizophrenia correctly? Schizophr Res (2011) 130:1–10. 10.1016/j.schres.2011.05.004 21645998 PMC3139794

[41] Ennis SR Ríos-Vargas M Albert NG The hispanic population: 2010. US Census Bureau: US Department of Commerce, Economics and Statistics Administration (2011).

[42] California Department of Education 2018-19 Enrollment by Ethnicity and Grade. (2018). Available at: https://dq.cde.ca.gov/dataquest/dqcensus/EnrEthGrd.aspx?cds =00&agglevel=State&year=2018-19.

[43] Aguilar-Gaxiola S Loera G Méndez L Sala M Nakamoto J Community-defined solutions for Latino mental health care disparities: California reducing disparities project, Latino strategic planning workgroup population report. UC Davis: Sacramento, CA (2012).

[44] Alderman T Addington J Bearden C Cannon TD Cornblatt BA Mcglashan TH Negative symptoms and impaired social functioning predict later psychosis in Latino youth at clinical high risk in the North American prodromal longitudinal studies consortium. Early Interv Psychiatry (2015) 9:467–75. 10.1111/eip.12128 PMC4362746 24576057

[45] Ventura J Subotnik KL Gitlin MJ Gretchen-Doorly D Ered A Villa KF Negative symptoms and functioning during the first year after a recent onset of schizophrenia and 8years later. Schizophr Res (2015) 161:407–13. 10.1016/j.schres.2014.10.043 PMC4308531 25499044

[46] Twamley EW Bengoetxea E Mondragon A Ojeda N Entrenamiento Cognitivo Compensatorio [Spanish translation of Compensatory Cognitive Training for Clients with Psychiatric Illness. (2012).

[47] Nuechterlein KH Green MF Kern RS Baade LE Barch DM Cohen JD The MATRICS consensus cognitive battery, part 1: test selection, reliability, and validity. Am J Psychiatry (2008) 165:203–13. 10.1176/appi.ajp.2007.07010042 18172019

[48] Mausbach BT Harvey PD Goldman SR Jeste DV Patterson TL Development of a brief scale of everyday functioning in persons with serious mental illness. Schizophr Bull (2007) 33:1364–72. 10.1093/schbul/sbm014 PMC2779885 17341468

[49] Harvey PD Green MF Nuechterlein KH Latest developments in the matrics process. Psychiatry (Edgemont) (2010) 7:49–52. PMC2898842 20622946

[50] Hall RCW Global assessment of functioning: a modified scale. Psychosomatics (1995) 36:267–75. 10.1016/S0033-3182(95)71666-8 7638314

[51] Weissman MM Bothwell S Assessment of social adjustment by patient self-report. Arch Gen Psychiatry (1976) 33:1111–5. 10.1001/archpsyc.1976.01770090101010 962494

[52] First M Spitzer R Gibbon M Williams J Structured clinical interview for DSM-IV-TR axis I disorders, research version, patient edition. SCID-I/P. (2002).

[53] Drake R Mueser K McHugo G Clinical Rating Scales. In: Sederer L Dickey B, editors. Outcomes assessment in clinical practice. Williams and Wilkins: Baltimore (1996).

[54] Bellack AS Gold JM Buchanan RW Cognitive rehabilitation for schizophrenia: problems, prospects, and strategies. Schizophrenia (1999) 25:257–74. 10.1093/oxfordjournals.schbul.a033377 10416730

[55] Spaulding WD Fleming SK Reed D Sullivan M Storzbach D Lam M Cognitive functioning in schizophrenia: implications for psychiatric rehabilitation. Schizophr Bull (1999) 25:275–89. 10.1093/oxfordjournals.schbul.a033378 10416731

[56] McGurk SR Meltzer HY The role of cognition in vocational functioning in schizophrenia. Schizophr Res (2000) 45:175–84. 10.1016/S0920-9964(99)00198-X 11042435

[57] Spaulding WD Reed D Sullivan M Richardson C Weiler M Effects of cognitive treatment in psychiatric rehabilitation. Schizophr Bull (1999) 25:657–76. 10.1093/oxfordjournals.schbul.a033409 10667738

[58] Seidman LJ Shapiro DI Stone WS Woodberry KA Ronzio A Cornblatt BA Association of neurocognition with transition to psychosis: Baseline functioning in the second phase of the north American prodrome longitudinal study. JAMA Psychiatry(2016), 1239–48. 10.1001/jamapsychiatry.2016.2479 PMC5511703 27806157

[59] Thakkar KN Park S Impaired passive maintenance and spared manipulation of internal representations in patients with schizophrenia. Schizophr Bull (2012) 38:787–95. 10.1093/schbul/sbq159 PMC3406515 21205676

[60] Clare L McKenna PJ Mortimer AM Baddeley AD Memory in schizophrenia: what is impaired and what is preserved? Neuropsychologia (1993) 31:1225–41. 10.1016/0028-3932(93)90070-G 8107983

[61] Kéri S Juhász A Rimanóczy Á Szekeres G Kelemen O Cimmer C Habit learning and the genetics of the dopamine D3 receptor: evidence from patients with schizophrenia and healthy controls. Behav Neurosci (2005) 119:687–93. 10.1037/0735-7044.119.3.687 15998189

[62] Perepletchikova F Kazdin AE Treatment integrity and therapeutic change: Issues and research recommendations. Clin Psychol Sci Pract (2005) 12:365–83. 10.1093/clipsy/bpi045

[63] Cohen J Statistical power analysis for the behavioural sciences. 2nd ed. L. Erlbaum Associates: Hillsdale, NJ (1998).

[64] Hedeker D Gibbons RD Waternaux C Sample size estimation for longitudinal designs with attrition: comparing time-related contrasts between two groups. J Educ Behav Stat (1999) 24:70–93. 10.3102/10769986024001070

[65] Kraemer HC Wilson GT Fairburn CG Agras WS Mediators and moderators of treatment effects in randomized clinical trials. Arch Gen Psychiatry (2002) 59:877–83. 10.1001/archpsyc.59.10.877 12365874

[66] Hooker CI Carol EE Eisenstein TJ Yin H Lincoln SH Tully LM A pilot study of cognitive training in clinical high risk for psychosis: initial evidence of cognitive benefit. Schizophr Res (2014) 314–6. 10.1016/j.schres.2014.05.034 PMC4384393 24954429

[67] Hodgkinson S Godoy L Beers LS Lewin A Improving mental health access for low-income children and families in the primary care setting. Pediatrics (2017) 139:e20151175. 10.1542/peds.2015-1175 27965378 PMC5192088

[68] Sentell T Shumway M Snowden L Access to mental health treatment by English language proficiency and race/ethnicity. J Gen Intern Med (2007) 289–93. 10.1007/s11606-007-0345-7 PMC2150610 17957413

[69] Isasi CR Rastogi D Molina K Health Issues in Hispanic/Latino Youth. J Lat Psychol (2016) 4:67–82. 10.1037/lat0000054.Health 27347457 PMC4915390

